# Stereotactic body radiation therapy for hepatocellular carcinoma: a comprehensive review

**DOI:** 10.3389/fonc.2026.1747449

**Published:** 2026-01-29

**Authors:** Yupeng Di, Gang Ren, Yingjie Wang, Lingling Meng, Jing Li

**Affiliations:** 1 Department of Radiation Protection Medicine, School of Preventive Medicine, Fourth Military Medical University, Xi’an, China; 2 Ministry of Education Key Lab of Hazard Assessment and Control in Special Operational Environment, Xi’an, China; 3 Department of Radiation Oncology, Air Force Medical Center, People’s Liberation Army (PLA), Beijing, China; 4 Department of Radiotherapy, Peking University Shougang Hospital, Beijing, China; 5 Department of Radiation Oncology, Senior Department of Oncology, The First Medical Center of People’s Liberation Army (PLA) General Hospital, Beijing, China

**Keywords:** efficacy, hepatocellular carcinoma, immunotherapy, stereotactic body radiation therapy, treatment outcomes

## Abstract

Hepatocellular carcinoma (HCC) presents significant challenges in modern surgical and radiation oncology, primarily due to biological heterogeneity and the complexities of underlying liver cirrhosis. This review evaluates stereotactic body radiation therapy (SBRT) as a mature, curative-intent modality within the multidisciplinary management of primary hepatic malignancies. Drawing parallels with high-dose-per-fraction successes in early-stage non-small cell lung cancer, pancreatic adenocarcinoma, and spinal metastases, we analyze the physical evolution, radiobiological principles, and functional dosimetric constraints specific to the hepatic microenvironment. Specifically, we examine advanced motion management, distinguishing pre-treatment setup from real-time intrafractional monitoring via kilovoltage projection streaming images (kV-PSI) and MR-guidance. We evaluate the prognostic value of metabolic imaging parameters, specifically *SUL_peak_* and radiomic signatures, alongside the therapeutic synergy between SBRT and systemic agents, including tyrosine kinase inhibitors (TKIs) and immune checkpoint inhibitors (ICIs). Additionally, the review explores the emerging role of artificial intelligence (AI) in automating planning and executing real-time online adaptive radiotherapy (ART). Finally, by synthesizing pathological evidence of complete necrosis in explanted specimens following conversion therapy, this work provides a roadmap for optimizing SBRT across the clinical spectrum of HCC.

## 1 Introduction

Hepatocellular carcinoma (HCC) is the leading cause of liver-related mortality and the third most common cause of cancer-related death globally. Managing HCC requires a careful balance due to the underlying liver pathology. Therapeutic decisions must weigh the goal of aggressive tumor eradication against the need to preserve cirrhotic parenchyma, which often has diminished functional and regenerative capacity. While radical resection and orthotopic liver transplantation remain the curative gold standards, only a minority of patients are eligible at initial diagnosis. Disqualification is often due to macrovascular involvement, anatomical constraints, or insufficient future liver remnant volume relative to tumor size.

Historically, liver tumors were considered largely unsuitable for external beam radiation therapy (EBRT). The liver is a radiosensitive organ, and older 2D or 3D conformal techniques often resulted in fatal radiation-induced liver disease (RILD), even at sub-therapeutic doses. However, technological advancements over the last decade have redefined the role of radiotherapy, mirroring paradigm shifts seen in early-stage lung cancer and spinal oligometastases. Stereotactic body radiation therapy (SBRT) has subsequently emerged as a critical treatment modality. SBRT delivers highly conformal, ablative doses of radiation—typically a biologically effective dose (BED_10_) of ≥100 Gy—in a limited number of fractions (usually 3 to 10). Its clinical success relies on a sharp dose fall-off at the interface between the target and normal tissue, sparing the functional liver reserve from toxic radiation levels.

Unlike thoracic applications, where lung density provides high contrast for tumor localization, liver SBRT requires advanced motion management. This is necessary to account for soft-tissue density similarities and the complex, non-linear respiratory motion of the abdomen. This review provides a comprehensive analysis of SBRT for HCC, focusing on foundational physics, synergies with systemic therapies, and the emerging role of conversion therapy as a bridge to curative intervention.

## 2 Basics of SBRT for HCC

### 2.1 Physics of conformality: photon precision and the proton frontier

The evolution of liver SBRT is characterized by a continuous drive toward steeper dose fall-off and enhanced target conformality. Building on principles established in early-stage NSCLC—where local control is correlated with dose density—Intensity-Modulated Radiation Therapy (IMRT) and Volumetric-Modulated Arc Therapy have become standard modalities for hepatic delivery (1, 2). These techniques utilize inverse planning algorithms to modulate thousands of beamlets, sculpting high-dose volumes around irregular tumors while sparing fragile central biliary tracts and adjacent gastrointestinal structures. This parallels the strict dosimetric requirements of pancreatic SBRT, where sub-millimeter precision is essential to avoid radiation-induced injury to the stomach and duodenum (3).

Beyond photon-based therapies, Proton Beam Therapy (PBT) represents a significant advancement in hepatobiliary oncology. Protons utilize the Bragg Peak effect, depositing maximum ionizing energy at a specific depth with virtually no exit dose. This physical characteristic facilitates the irradiation of large liver tumors (often >10 cm in diameter), which might be unsuitable for photon therapy due to the excessive “low-dose bath” delivered to the remaining healthy parenchyma. In patients with underlying cirrhosis, where the threshold for RILD is reduced, these physical advantages may prevent cumulative functional decline. Dosimetric studies suggest that for tumors adjacent to the heart, PBT offers superior sparing of low-to-intermediate dose volumes, potentially mitigating late-term toxicity (4, 5).

### 2.2 Intrafraction motion management and the interplay effect

Safe SBRT delivery in mobile organs requires distinguishing between pre-treatment setup verification and real-time intrafraction monitoring. In both lung and liver tumors, respiratory motion is frequently non-linear and subject to baseline drifts ( >5 mm during a single fraction). While Cone-beam CT (CBCT) provides a static volumetric image prior to delivery, it cannot fully account for the “interplay effect”—a dosimetric error caused by the interference between the motion of the multi-leaf collimator and tumor movement, potentially leading to dose heterogeneity (6).

Technological advancements addressing this include kV-PSI. As described by Katano et al., this system utilizes a high transfer rate ( >10 Hz) to register internal diaphragmatic landmarks against digitally reconstructed radiographies in real-time (4). This enables the radiation beam to track or gate the tumor with sub-millimeter precision, eliminating the need for invasive fiducial markers—a critical safety consideration for patients with coagulopathy. Furthermore, Magnetic Resonance-guided Linear Accelerators allow for continuous soft-tissue visualization during beam delivery, providing superior contrast for tumors that are poorly visualized on standard X-ray systems.

### 2.3 Respiratory motion management strategies

The choice between Breath-hold (BH) and Free-breathing (FB) protocols depends on clinical feasibility and patient functional reserve. Deep Inspiration Breath-hold (DIBH), adapted from breast cancer SBRT, is used to displace the target volume away from critical structures like the heart (7). In HCC, DIBH stabilizes the tumor and increases separation between the hepatic dome and the stomach. Alternatively, Exhalation Breath-hold may be utilized, as the diaphragm position is often most reproducible at end-expiration.

However, patients with advanced HCC often present with ascites or poor pulmonary compliance, precluding the use of repeated BH cycles. For these cohorts, FB SBRT combined with abdominal compression or respiratory gating serves as a viable alternative. Abdominal compression effectively reduces motion amplitude ( <5 mm) but must be balanced against patient comfort and organ deformation. Clinical data indicate that objective response rates in macrovascular compartments—particularly Portal Vein Tumor Thrombosis (PVTT)—are significantly improved when strict motion management strategies are enforced (8). The integration of real-time monitoring with respiratory stabilization ensures that the high-gradient dose distribution planned on static CT simulation is accurately delivered to the dynamic target.

### 2.4 Dosimetric principles and hepatic reserve protection

Liver SBRT planning is guided by radiobiological modeling. To maximize Tumor Control Probability (TCP), the Biologically Effective Dose ( $BED_{10}$) typically exceeds 100 Gy (9, 10). Simultaneously, the Normal Tissue Complication Probability (NTCP) for RILD must be minimized. For Child-Pugh A patients, a common regimen is 45–50 Gy in 3–5 fractions (assuming an α/β ratio of 10 for tumor). Conversely, for Child-Pugh B patients with compromised regenerative capacity, dose de-escalation or moderate hypofractionation (e.g., 40–50 Gy in 5–10 fractions) is utilized to prevent hepatic failure.

Current consensus indicates that the volume of normal liver receiving <15 Gy ( $V_{<15\text{ Gy}}$) is a superior predictor of RILD compared to mean liver dose (MLD) in cirrhotic patients (11). Additionally, sparing the central hepatobiliary tract is paramount to preventing biliary strictures, with standard constraints typically limiting the high-dose volume significantly (12). Emerging functional dosimetry protocols, utilizing SPECT/CT, enable “regional sparing” by prioritizing the protection of metabolically active parenchyma over purely anatomical volumes (13, 14).

## 3 Epidemiology and pathophysiology of HCC

### 3.1 The shifting global paradigm of risk factors

HCC constitutes a substantial and growing global health burden, with incidence trending upward across numerous regions. The global incidence of HCC is driven by diverse risk factors that vary significantly among populations. Historically, chronic hepatitis B virus (HBV) and hepatitis C virus (HCV) infections were the primary established risk factors. In Asia and sub-Saharan Africa, HBV remains the predominant etiology, accounting for the majority of HCC cases. For example, in Mozambique, a region with exceptionally high prevalence, clinical assessments have shown that 90% of HCC patients test positive for the virus (15). The Global Burden of Disease Study 2017 reported nearly 841,000 newly diagnosed liver cancer cases and over 781,000 deaths annually, more than double the numbers recorded in 1990, with viral hepatitis identified as the leading cause (16).

However, the epidemiological landscape is undergoing a significant transition. Non-alcoholic fatty liver disease (NAFLD) and its aggressive manifestation, non-alcoholic steatohepatitis (NASH), are emerging as dominant risk factors, particularly in Western nations with a high prevalence of obesity and metabolic syndrome (17). Malignant progression in NAFLD is more likely in individuals harboring specific genetic predispositions—such as the *PNPLA3* gene polymorphism—which exacerbates the inflammatory transition to cirrhosis. Other contributors include excessive alcohol consumption, aflatoxin-B1, and pesticide exposure. Interestingly, studies in Thailand identified shared risk factors between HCC and intrahepatic cholangiocarcinoma, finding that metabolic comorbidities significantly increased individual risk (18). Furthermore, patients with underlying chronic liver disease are at heightened risk of developing second primary malignancies, necessitating prolonged surveillance (19).

### 3.2 Molecular pathways and genomic regulation

HCC development is a complex process involving the sequential dysregulation of multiple molecular pathways. One of the pivotal axes implicated in hepatocarcinogenesis is the PI3K/AKT signaling pathway. Research investigating AT-rich interaction domain 4B in HCC has revealed that this protein may act as a critical activator of this pathway (20). In HCC cohorts undergoing resection, high ARID4B expression was significantly associated with poor tumor differentiation, microvascular invasion, and shortened disease-free survival. Molecular knockdown of ARID4B in human cell lines has been shown to suppress the PI3K/AKT axis, leading to a marked reduction in cell proliferation and invasive potential.

The Sonic Hedgehog signaling pathway also plays a decisive regulatory role in HCC pathogenesis, involving various aspects of tumor growth, invasion, and tumor microenvironment (TME) modulation. The prevalence of SHH ligands in approximately 50% of HCC tissues underscores its importance in mediating resistance to chemotherapy and targeted therapies (21). Epigenetic modifications, particularly the hypermethylation of the promoter of hepatocellular carcinoma suppressor 1, further contribute to disease progression. The frequency of HCCS1 promoter methylation in HCC is significantly higher than in patients with chronic hepatitis B or healthy controls, correlating directly with advanced TNM stage (22).

### 3.3 The tumor microenvironment and radiobiological response

The TME significantly dictates the response of HCC to radiation therapy. The TME is a complex ecosystem comprising various cell types, including tumor-associated macrophages (TAMs). Intratumoral TAMs are consistently associated with adverse clinicopathological factors and poor prognosis. The CCL2/CCR2/Erk signaling pathway, activated through the interaction between HCC cells and TAMs, contributes to malignant progression; targeted inhibition of this axis could enhance SBRT efficacy (23).

Moreover, the TME modulates the systemic immune response to radiation. Radiation-induced lymphopenia is a concern associated with worse outcomes, a phenomenon also observed in lung and pancreatic cancers. Patients who develop acute severe lymphopenia exhibit significantly poorer overall survival. Interestingly, high pre-radiotherapy interleukin-7 levels have been identified as an independent predictor of reduced ASL risk, indicating that the baseline immune state is a critical determinant of radiotherapy tolerance (24). Furthermore, hypoxia within the TME significantly reduces radiation effectiveness by limiting the production of reactive oxygen species (ROS). Strategies aimed at mitigating hypoxia, such as anti-angiogenesis agents, may “normalize” aberrant tumor vasculature, thereby improving oxygen delivery and increasing the susceptibility of HCC cells to SBRT (25).

## 4 Diagnostic and imaging techniques in HCC

### 4.1 Advances in morphological and functional imaging modalities

The diagnosis and staging of HCC have been revolutionized by advanced imaging technologies, which are pivotal for detection and therapeutic monitoring. Ultrasound remains the primary screening tool, while contrast-enhanced ultrasound shows promise in characterizing nodules; arterial phase hyperenhancement followed by Kupffer phase hypoenhancement reliably indicates moderate differentiation (26). CT and MRI are indispensable for detailed characterization. Dynamic contrast-enhanced protocols demonstrate the characteristic “wash-in and wash-out” vascular patterns. MRI, particularly with hepatocyte-specific contrast media, demonstrates higher sensitivity than CT for detecting small lesions ( <2 cm), although sensitivities for larger focal lesions are comparable (27).

Furthermore, PET/CT utilizing radiopharmaceuticals like ^11^C-choline has been investigated for staging and detecting extrahepatic disease. Fusing PET metabolic data with morphological CT/MRI findings yields the highest diagnostic accuracy (28). However, the most critical advancement for SBRT monitoring is the implementation of ^18^F-FDG PET/CT. Although historical views questioned the FDG avidity of HCC, metabolic parameters are effectively used to assess aggressive phenotypes and evaluate PVTT.

### 4.2 Metabolic response assessment: *SUL_peak_*versus *SUV_max_*

A critical challenge in SBRT for HCC is assessing response within the vascular compartment. Unlike primary lesions, which may undergo slow cystic degeneration, metabolic activity in PVTT more accurately indicates viability. Quantitative metabolic parameters, specifically *SUL_peak_*(Peak Standardized Uptake Value corrected for lean body mass), are increasingly favored over traditional *SUV_max_*. HCC patients often suffer from cirrhosis-related sarcopenia or nutritional deficiencies; thus, *SUL_peak_* provides a more homogeneous measurement of metabolic flux per unit of active tissue.

Sahai et al. demonstrated that significant *SUL_peak_* reduction at 3 months post-SBRT (aligned with PERCIST criteria) is a powerful independent predictor of prolonged overall survival (8). Since SBRT can induce localized inflammatory changes that mimic persistent disease on CT or *SUV_max_*, utilizing *SUL_peak_* enables a “metabolic recalibration” that accurately distinguishes therapeutic success from persistent tumor viability. This mirrors response monitoring in pancreatic SBRT, where tumor shrinkage is often minimal despite successful internal sterilization.

### 4.3 Imaging in precision treatment planning

Imaging is paramount in the SBRT planning process, facilitating target definition and the assessment of relationships between the tumor and organs at risk (OARs). Gadoxetate disodium-enhanced MRI is particularly effective for delineating functional liver tissue. By employing IMRT techniques guided by EOB-MRI, clinicians can spare functional sub-regions, reducing mean dose to the active parenchyma while delivering ablative doses to the target (14).

Four-dimensional computed tomography accounts for respiratory motion by acquiring images across different breathing phases. This allows for precise definition of the internal target volume (ITV), a technique optimized in lung SBRT to ensure the moving tumor remains within the high-dose volume (29). During delivery, systems like CBCT verify patient positioning. Experience from lung SBRT emphasizes that bony anatomy matching is insufficient for the liver, necessitating tumor-specific or surrogate-based image guidance to maintain a precise planning target volume (PTV) margin (30).

### 4.4 Biomarkers and kinetic monitoring

Biomarkers hold significant potential to enhance early diagnosis and prognostication. Alpha-fetoprotein (AFP) is the established standard, but its sensitivity is often limited. Complementary biomarkers, such as proteasome and sICAM-1, improve diagnostic performance when combined with AFP (31). MicroRNAs have also emerged as promising circulating markers; a meta-analysis revealed that miR-21 possesses the highest diagnostic efficiency among these markers (32). Similarly, plasma levels of lncRNAs, such as HULC and Linc00152, are significantly upregulated in HCC patients and can accurately discriminate between patients and healthy controls (33).

Longitudinally, the kinetics of markers like PIVKA-II are particularly relevant for SBRT monitoring. Success in treating PVTT is often reflected by a rapid PIVKA-II decline within three months of radiation completion, providing a biochemical correlate to metabolic responses seen on ^18^F-FDG PET/CT (8). These biomarkers provide a non-invasive framework for real-time monitoring and personalized therapy adjustments.

## 5 Clinical outcomes and treatment strategies

### 5.1 Clinical efficacy of SBRT monotherapy

SBRT has demonstrated high clinical efficacy in managing HCC, serving as a potent alternative for patients ineligible for surgical resection. Numerous prospective and retrospective investigations report consistently high local control rates, often exceeding 85% at one year, coupled with acceptable toxicity profiles. Individual outcomes and treatment characteristics of representative clinical trials are summarized in **Table 1**. In a pivotal study involving inoperable lesions, actuarial LC rates at 6, 12, and 24 months were consistently robust. Significant prognostic factors for local efficacy typically include biological effective dose ( $BED_{10}$) and initial tumor volume, mirroring the dose-response relationship established in early-stage NSCLC where dose density drives sterilization (9).

**Table 1:** Summary of representative SBRT studies for HCC.

Study (Year)	Patient	Dose/Fractionation	Local control	Overall survival	Key focus
Kwon et al. (2010) (53)	42	30–39 Gy/3f	1-yr: 59.6% (CR)	1-yr: 92.9%	Solitary lesions
Su et al. (2016) (35)	132	High Dose (Diverse)	1-yr: 90.9%	5-yr: 64.3%	Small HCC
Sahai et al. (2025) (1)	24	12-31.5 Gy/3-7f	DCR: 50%	Median: 10.9 m	PVTT/Multimodality
Jeong et al. (2021) (37)	100+	BH vs FB	Comparable	Comparable	SBRT vs RFA

HCC, Hepatocellular carcinoma; SBRT, Stereotactic body radiation therapy; Gy, Gray; f, Fractions; yr, Year; CR, Complete response; OS, Overall survival; LC, Local control; DCR, Disease control rate; m, Months; PVTT, Portal vein tumor thrombosis; BH, Breath-hold; FB, Free-breathing; RFA, Radiofrequency ablation.

Another substantial cohort treated for inoperable HCC showed remarkable 1-year and 2-year LC rates, with corresponding overall survival rates reflecting the underlying disease extent. Multivariable analyses frequently identify sex, Child-Pugh class, and treatment history as independent predictors of long-term survival (34). Furthermore, in the Chinese experience with small primary or recurrent HCC, SBRT achieved high long-term control. The presence of multiple nodules and Child-Pugh B classification were associated with poorer progression-free survival (PFS), emphasizing that while SBRT is physically capable of ablating multiple targets, biological liver function remains the ultimate limiting factor (35).

### 5.2 SBRT versus other locoregional modalities

When situating SBRT within the hierarchy of interventions, comparisons with radiofrequency ablation and transarterial chemoembolization are critical. A retrospective analysis from the National Cancer Database initially favored RFA; however, subsequent studies utilizing inverse probability of treatment weighting for small tumors ( <3 cm) demonstrated that SBRT and RFA provide comparable outcomes regarding LC and intrahepatic recurrence-free survival (36, 37). Crucially, SBRT offers a technical advantage over RFA by avoiding the “heat-sink effect.” While RFA efficacy is frequently compromised near large hepatic vessels where blood flow dissipates thermal energy, SBRT is unaffected by local perfusion, making it preferred for tumors adjacent to major vasculature.

SBRT also differs fundamentally from TACE. While TACE is primarily an embolic and cytotoxic approach suitable for multifocal disease, SBRT delivers a definitive ablative dose. Propensity-score matching comparing TACE plus thermal ablation versus TACE plus SBRT for inoperable HCC suggested potential survival benefits for T-TA in specific subgroups; however, T-SBRT was associated with a distinct toxicity profile (38). Unlike spinal metastasis management where stabilization is the goal, SBRT in HCC aims for biological cure, conceptually aligned with its use as a “definitive” treatment for NSCLC in borderline surgical candidates (39).

### 5.3 Conversion therapy and pathological complete necrosis

Perhaps the most transformative development is the emergence of SBRT as a catalyst for conversion therapy. For patients presenting with advanced PVTT, historically considered terminal, SBRT acts as a bridge to downstaging and potential cure. Sahai et al. documented the potential of multimodality protocols to bridge previously inoperable patients to living donor liver transplantation (LDLT).

In these cohorts, patients exhibiting significant metabolic response—defined by reduction per PERCIST criteria—underwent radical surgery after downstaging. Pathological examination of explanted livers in several cases revealed 100% pathological complete necrosis (pCN) in both the primary tumor and vascular thrombus (1, 8). This represents a paradigm shift: transforming terminal, vascular-invasive disease into a surgically curable state through high-precision physics. Achieving pCN validates the ablative power of doses ( $BED_{10}>100\text{ Gy}$) and demonstrates SBRT’s ability to eradicate microscopic disease within the vascular intima.

### 5.4 Synergy in combination therapies

Combination therapies involving SBRT and systemic agents are redefining survival curves. The synergy between SBRT and immunotherapy is predicated on inducing immunogenic cell death (ICD) and releasing neoantigens, which can activate a systemic anti-tumor response known as the abscopal effect. Preclinical models have shown that concurrent antibody delivery and SBRT significantly increase cytotoxic T cell infiltration within the TME, leading to improved survival (40).

Furthermore, combining SBRT with TACE for patients with macroscopic vascular invasion has shown superior response rates compared to sorafenib monotherapy, with a hazard ratio (HR) favoring the combination (41). A milestone in clinical evidence is the NRG/RTOG 1112 trial, which demonstrated that adding SBRT to sorafenib significantly improved OS, PFS, and time to progression compared to sorafenib alone in advanced HCC (42). Other investigative avenues include using PARP inhibitors as radiosensitizers to sensitize HCC by interfering with DNA damage response pathways, a strategy showing promise in pancreatic cancer.

## 6 Controversies, challenges, and future directions

### 6.1 Optimal fractionation and dose escalation

Determining the optimal dose and fractionation schedule for liver SBRT remains a subject of intense debate. While modern linear accelerators can physically deliver extremely high doses, the biological tolerance of the host liver remains the primary limiting factor. Various regimens are utilized globally, ranging from 48–60 Gy in 3 fractions to more protracted schedules of 50–60 Gy in 5–10 fractions.

The logic of fractionation in the liver parallels spinal SBRT, where spinal cord tolerance dictates dose feasibility (11). In HCC, the critical organ at risk (OAR) is the functional liver reserve. Hypofractionated schedules (e.g., 1–3 fractions) may be biologically more potent against the tumor but carry a higher risk of triggering RILD in patients with cirrhosis. Conversely, increasing the number of fractions may allow for better parenchymal repair but could inadvertently reduce the biologically effective dose ( $BED_{10}$) and the probability of achieving pCN.

### 6.2 Managing RILD and functional dose constraints

RILD remains the most concerning toxicity in hepatic SBRT. Historically, RILD risks nearly marginalized radiotherapy for HCC. Modern strategies focus on both morphological and functional constraints. A significant shift in clinical practice is the adoption of the Albumin-Bilirubin (ALBI) grade, which provides a more objective assessment of liver function than the traditional Child-Pugh classification, allowing for precise dose adaptation (43).

To manage RILD risk, using NTCP models is crucial. Modern protocols utilize parameters such as $V_{eff}$ or MLD to ensure safety. Functional imaging, particularly ^99m^Tc-sulfur colloid SPECT/CT, allows clinicians to distinguish between vascularized, metabolic parenchyma and “dead” cirrhotic regions. This enables functional-sparing plans that reduce the probability of post-radiotherapy decompensation (13). Furthermore, advanced motion management effectively reduces the PTV margin, sparing larger volumes of healthy liver.

### 6.3 Artificial intelligence and technological innovation

The future of HCC management is linked to technological innovation, with Artificial Intelligence (AI) and machine learning transforming workflows. Deep learning algorithms are being trained to automate the auto-contouring of the liver and OARs, facilitating rapid planning (44). AI-driven segmentation achieves accuracy comparable to expert radiologists, significantly reducing “time-to-beam” for acute cases.

Furthermore, AI enables Online Adaptive Radiotherapy (ART). Since internal abdominal anatomy, including gastric and intestinal filling, changes daily, the “plan of the day” requires adjustment. Online ART utilizes rapid re-calculation and deformable image registration (DIR) to adjust the plan while the patient is on the treatment table. This ensures that the high-dose ablative peak remains centered on the tumor while accounting for minute-to-minute shifts in sensitive OARs like the duodenum (45).

### 6.4 Radiomics, liquid biopsies, and personalized medicine

The integration of radiomics and liquid biopsies represents the final component of personalized medicine. Radiomics utilizes AI to extract high-dimensional textural features from pre-treatment CT/MRI that are imperceptible to the human eye. These radiomic signatures, such as SAMSN1 expression, can predict tumor radiosensitivity and individual RILD risk with higher accuracy than morphological scores alone (46).

Simultaneously, sequencing cell-free DNA from plasma—a “liquid biopsy”—allows for real-time monitoring of genetic mutations and treatment response without invasive tissue biopsies (47–49). This enables dynamic therapy adjustment, such as optimizing the timing of immune checkpoint inhibitors to maximize the abscopal effect and overcome treatment resistance (50–52).

## 7 Conclusion

SBRT has transitioned from an experimental alternative to a cornerstone of multidisciplinary HCC management. Its evolution is defined by the convergence of high-precision dosimetry, advanced understanding of the TME, and state-of-the-art diagnostic monitoring. By integrating technological lessons from lung and spinal SBRT, clinicians have refined the therapeutic ratio for HCC, achieving local control rates comparable to surgical resection.

A critical impact of SBRT lies in its role as a catalyst for conversion therapy, providing a curative pathway for advanced HCC with PVTT. In cases previously deemed palliative, SBRT facilitates the achievement of pCN. Moving forward, the integration of AI, functional dosimetry, and next-generation systemic agents will further enhance the precision and safety of SBRT, particularly for patients with compromised hepatic reserve. As oncology moves toward a personalized framework, SBRT will remain at the forefront—not merely as a local therapy, but as a biological bridge and systemic partner in the comprehensive treatment of HCC.
